# Acquired resistance to zoledronic acid and the parallel acquisition of an aggressive phenotype are mediated by p38-MAP kinase activation in prostate cancer cells

**DOI:** 10.1038/cddis.2013.165

**Published:** 2013-05-23

**Authors:** M R Milone, B Pucci, F Bruzzese, C Carbone, G Piro, S Costantini, F Capone, A Leone, E Di Gennaro, M Caraglia, A Budillon

**Affiliations:** 1 Centro di Ricerche Oncologiche di Mercogliano (CROM), Mercogliano (AV), Italy; 2 Experimental Pharmacology Unit, Department of Research, IRCCS-Istituto Nazionale Tumori G Pascale, Naples, Italy; 3 Department of Biochemistry and Biophysics, Second University of Naples, Naples, Italy

**Keywords:** P38-MAPK, drug resistance, zoledronic acid, prostate cancer

## Abstract

The nitrogen-containing bisphosphonates (N-BP) zoledronic acid (ZOL) inhibits osteoclast-mediated bone resorption, and it is used to prevent skeletal complications from bone metastases. ZOL has also demonstrated anticancer activities in preclinical models and, recently, in cancer patients, highlighting the interest in determining eventual mechanisms of resistance against this agent. In our study, we selected and characterised a resistant subline of prostate cancer (PCa) cells to better understand the mechanisms, by which tumour cells can escape the antitumour effect of ZOL. DU145R80-resistant cells were selected in about 5 months using stepwise increasing concentrations of ZOL from DU145 parental cells. DU145R80 cells showed a resistance index value of 5.5 and cross-resistance to another N-BP, pamidronate, but not to the non-nitrogen containing BP clodronate. Notably, compared with DU145 parental cells, DU145R80 developed resistance to apoptosis and anoikis, as well as overexpressed the anti-apoptotic protein Bcl-2 and oncoprotein c-Myc. Moreover, DU145R80 cells underwent epithelial to mesenchymal transition (EMT) and showed increased expression of the metalloproteases MMP-2/9, as well as increased invading capability. Interestingly, compared with DU145, DU145R80 cells also increased the gene expression and protein secretion of VEGF and the cytokines Eotaxin-1 and IL-12. At the molecular level, DU145R80 cells showed strong activation of the p38-MAPK-dependent survival pathway compared with parental sensitive cells. Moreover, using the p38-inhibitor SB203580, we completely reversed the resistance to ZOL, as well as EMT marker expression and invasion. Furthermore, SB203580 treatment reduced the expression of VEGF, Eotaxin-1, IL-12, MMP-9, Bcl-2 and c-Myc. Thus, for the first time, we demonstrate that the p38-MAPK pathway can be activated under continuous extensive exposure to ZOL in PCa cells and that the p38-MAPK pathway has a critical role in the induction of resistance, as well as in the acquisition of a more aggressive and invasive phenotype.

Prostate cancer (PCa) is the second most common cancer in men, accounting for 10% of male cancers.^1^ Despite continuing efforts to develop effective and safe chemotherapeutic, targeted and hormonal therapies, the overall survival for patients with locally advanced, recurrent or metastatic disease, has not improved significantly. Indeed, although chemotherapy remains the main treatment option for androgen-refractory metastatic prostate cancer (CRPC), resistance occurs in half of all patients and inevitably develops even in those who initially respond.^2^

About 90% of PCa metastatic patients will develop bone metastases that are primarily responsible for patient morbidity and mortality. Proliferation of PCa cells in the bone marrow induces osteoclast activation and bone resorption, both of which are also necessary for tumour growth and clonal expansion through a complex interplay between tumour cells, bone marrow and associated growth factors and cytokines.^3^

Nitrogen-containing bisphosphonates (N-BPs), such as zoledronic acid (ZOL), inhibits bone resorption by disrupting osteoclast function and survival, and ZOL is currently used in oncological practice to reduce skeletal complications and pain related with bone metastasis of several neoplasms, including PCa.^3, 4^ Accumulated evidence indicates that N-BPs, including ZOL, may also exert potent antitumour effects in several cancer types, including PCa.^5, 6, 7^ Several clinical trials have shown that ZOL may improve survival and extend the time to progression and/or exert an antitumour effect in cancer patients.^3^ ZOL might have a beneficial antitumour effect that could occur, indirectly creating a less favourable microenvironment for the survival of metastatic tumour cells, including immune modulation. Alternatively, or perhaps in addition, ‘direct' effects such as an induction of tumour cell apoptosis, a reduction in proliferation rates and tumour angiogenesis, and potential synergistic effects with anti-cancer therapies might be clinically important.^3, 8^

N-BPs inhibit farnesylpyrophosphate synthase (FPPS), a key enzyme of the mevalonate pathway. This event causes farnesylpyrophosphate (FPP) and geranylgeranyl pyrophosphate (GGPP) deprivation with a consequent failure of farnesylation and geranylation of small GTPases of the RAS superfamily, thereby disrupting the subcellular localisation and normal function of these essential signalling proteins and eventually stopping osteoclast-mediated bone resorption.^8, 9^ These properties also represent the molecular mechanism of the putative direct antitumour effect of N-BPs because the mevalonate pathway has been implicated in various aspects of tumour development and progression.^10^

Despite the importance of ZOL in the clinical management of cancer, few studies have reported the molecular mechanisms underlying the development of ZOL resistance in cancer cells.^11, 12, 13^

In our study, we have selected and characterised for the first time a ZOL-resistant PCa cell line. Notably, in addition to specific resistance to the antiproliferative and pro-apoptotic effects of ZOL, this cell line acquired a very aggressive phenotype that is characterised by resistance to anoikis, increased invasive capability and epithelial to mesenchymal transition (EMT). Both the ZOL resistance and aggressive phenotype appeared to be mediated by strong activation of the p38-MAPK-dependent pathway, and we show that it is possible to overcome the resistance and aggressiveness with a specific inhibitor of p38-MAPK.

## Results

### Development of the ZOL-resistant PCa subline DU145R80

**Figure 1:**
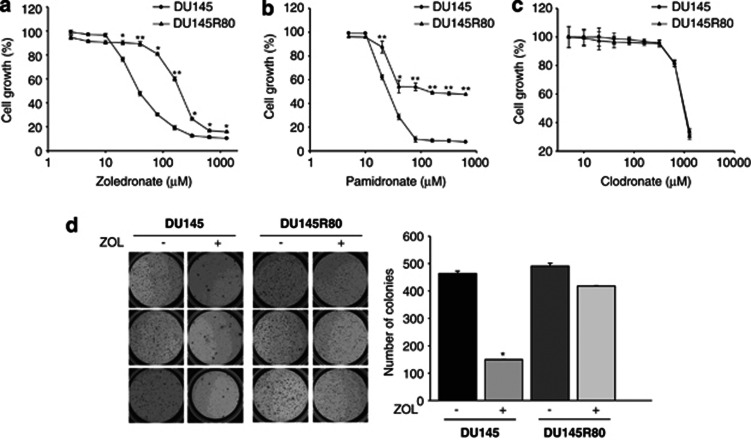
Development of ZOL-resistant DU145R80 PCa cell line. DU145 sensitive and DU145R80 resistant cells were treated with increasing concentrations of (**a**) ZOL, (**b**) Pamidronate or (**c**) Clodronate, for 96 h and cell growth assessment was done by sulforhodamine B colorimetric assay (see Materials and Methods). Cell growth is expressed as percentage of control for each time-point. Values are the mean±S.D. from at least three independent experiments performed in quadruplicates (**P⩽0.001 and *P⩽0.05, DU145R80 *versus* DU145). (**d**) Soft-agar clonogenic assay was performed on DU145 and DU145R80 cells untreated or treated with ZOL (20 *μ*ℳ) for 14 days, in 24-well plates. Colonies >100 *μ*m were scored by a colony counter. Left: images from a representative experiment; right: values expressed as number of colonies are means±S.D. from at least two independent experiments performed in triplicates. Statistical analysis demonstrated significant differences only in DU145 cells (*P<0.001, ZOL *versus* untreated)

To better investigate the mechanisms by which tumour cells can escape the antitumour effect of ZOL, we have selected the DU145R80 PCa-resistant cell model in about 6 months from parental DU145 cells by stepwise exposure to increasing concentrations of ZOL (until 80 *μ*ℳ). The drug-adapted DU145R80 cell line showed a significantly higher IC_50_ compared with parental DU145 cells (145.90±1.8 *versus* 22.99±0.2, respectively; *P*<0.001), resulting in more than fivefold resistance to ZOL (resistance index (RI)=5,5) (Figure 1a). Interestingly, the resistant cells showed a reduced proliferation rate compared with parental DU145 cells (Supplementary Figure 1). To confirm that the resistance to ZOL was restricted to N-BPs, we evaluated also the effect of clodronate, a non-N-BP, or pamidronate, another N-BP (Figures 1b and c). The results suggested cross-resistance to pamidronate (IC_50_ DU145R80=186.15±5.3 *versus* DU145=25.67±2.3; *P*<0.001) (RI=7.2) but not to clodronate (RI=1), confirming a specific mechanism of resistance to N-BP agents. Finally, we confirmed the higher resistance of DU145R80 to ZOL compared with parental DU145 cells under anchorage-independent conditions (Figure 1d).

### DU145R80 cells acquired resistance to spontaneous apoptosis under adhesion and anoikis conditions and overexpressed Bcl-2 and c-Myc

**Figure 2:**
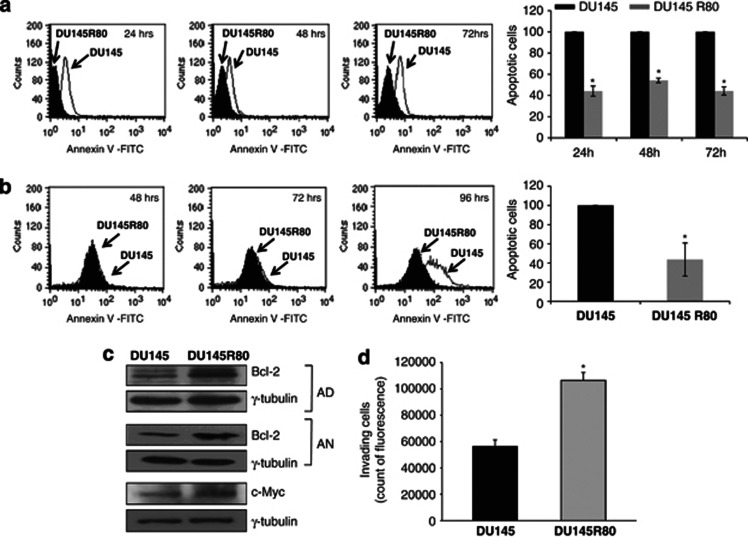
Resistance to apoptosis and anoikis and increased invasiveness of DU145R80 compared with DU145 cells. Apoptosis evaluated by AnnexinV binding and cytofluorimetric analysis in DU145 compared with DU145R80 cells, in adherent conditions (**a**), and non-adherent conditions (anoikis) (**b**), for the indicated time points (see Materials and Methods). Left: representative experiment; right: values of DU145R80 apoptotic cells expressed as per cent of DU145 parental cells (100%). Values are means±S.D. of three independent experiments. Statistical analysis demonstrated significant differences for all time points in adherent conditions (*P=0.007, 24 h; *P*=0.002, 48 h; *P*=0.005, 72 h) and at 96 h in non-adherent conditions (*P=0.031). (**c**) Western blot analysis of Bcl-2 in adherent (AD) and in anoikis conditions (AN) and of c-Myc. Cell lysates were obtained after 48 h of cell culture. *γ*-tubulin was used as protein loading control. (**d**) Invasion capability of DU145 and DU145R80 cells was evaluated as the ability to invade matrigel coated chambers (see Materials and Methods). Values are means±S.D. from three independent experiments performed in duplicates and statistical analysis is reported (*P=0.023, DU145R80 *versus* DU145 cells)

Resistance to anticancer drugs is often related to activation of prosurvival/antiapoptotic pathways. Using AnnexinV staining and cytofluorimetric analysis of adherent cells performed at 24, 48 or 72 h after seeding, we demonstrated in DU145R80 cells a statistically significant reduction of >50% in the number of cells undergoing spontaneous apoptosis compared with DU145 parental cells at all time points examined (Figure 2a). Conversely, suppression of apoptosis during carcinogenesis is thought to have a central role in the development and progression of cancer. In particular, resistance to anoikis, defined as apoptosis induced by inadequate or inappropriate cell-matrix interactions, is emerging as a hallmark of metastatic cancer cells as it is critical for their survival after detachment and facilitates migration and reattachment at the metastatic niche.^14^ As shown in Figure 2b, the apoptotic rate of DU145R80 cells was dramatically lower compared with that of parental cells after 96 h of culture under anoikis conditions.

Accordingly, with the reduction of apoptotic events, we found overexpression of the antiapoptotic protein Bcl-2 in DU145R80 cells compared with DU145 parental cells under both adherent and anoikis growth conditions (Figure 2c). Interestingly, in DU145R80 cells, we also found a slight overexpression of the proto-oncogene c-Myc (Figure 2c), a protein involved in cell proliferation, angiogenesis, apoptosis and differentiation.^15^

### DU145R80 cells showed an invasive and more aggressive phenotype

**Figure 3:**
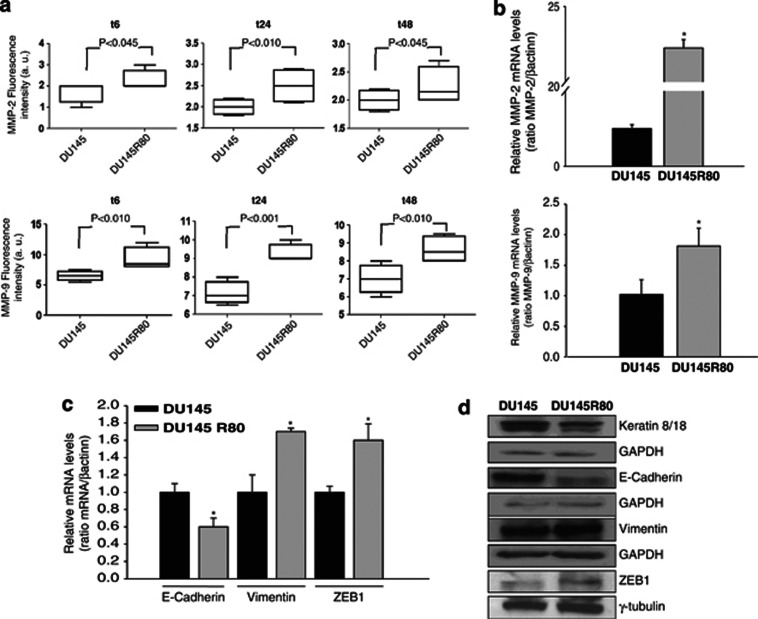
Increased expression of MMPs and EMT features in DU145R80 compared with DU145 cells. (**a**) MMP-2 and MMP-9 concentrations were evaluated in the culture media from DU145 and DU145R80 cells at the indicated time points after seeding by a multiplex ELISA-based immunoassay assay, and were plotted with box-and-whisker graphs. The boxes extend from the 25th to the 75th percentile and the line in the middle is the median. The error bars extend down to the lowest value and up to the highest. (**b**) MMP-2 and MMP-9 mRNA expression was evaluated by RT-PCR after 24 and 6 h of cell culture, respectively. The data are representative of at least three independent experiments, include the means±S.D. of technical triplicates and reported statistical analysis of DU145R80 *versus* DU145 cells (*P<0.001, MMP-2; *P*=0.045, MMP-9). (**c**) E-cadherin, Vimentin and ZEB1 mRNA expression was evaluated by RT-PCR after 24 h of cell culture. The data are representative of at least three independent experiments, include the means±S.D. of technical triplicates and reported statistical analysis of DU145R80 *versus* DU145 cells (*P=0.02, E-cadherin; *P*=0.007, Vimentin; *P*=0.005, ZEB1). (**d**) Western blot analysis of Keratin 8/18, E-cadherin, Vimentin and ZEB1 proteins expression evaluated after 48 h of cell culture. GAPDH and *γ*-tubulin were used as protein loading control

On the basis of previous data, we next examined whether DU145R80 cells, although their growth rate is lower compared with parental cells (Supplementary Figure 1), demonstrated a proinvasive phenotype. As shown in Figure 2d, we observed an enhanced ability of DU145R80 cells to invade matrigel-coated trans wells compared with DU145 cells. In agreement with this effect we also observed a statistically significant higher secretion of metalloproteinases 2 (MMP-2) and 9 (MMP-9) in the culture medium of DU145R80 cells at 6, 24 or 48 h after seeding, compared with parental cells (Figure 3a), and this result was confirmed by a concomitant increase in the gene expression of both MMPs (Figure 3b).

The EMT in cancer cells is associated with increased mobility, invasive phenotype and drug resistance and involves loss or lowered expression of epithelial markers (e.g., E-cadherin and keratins) and increased expression of mesenchymal markers (e.g., vimentin).^16^ As shown in Figure 3c, we demonstrated in DU145R80, compared with DU145 cells, a clear reduction of the E-cadherin mRNA level, and a concomitant upregulation of its repressor factor ZEB1, confirmed by analysis of protein expression (Figure 3d). Moreover, downregulation of additional epithelial markers, such as keratins 8/18, and upregulation of the mesenchymal marker vimentin confirmed an EMT transition in the resistant cell line (Figures 3c and d).

### DU145R80 cells increased the secretion of VEGF, Eotaxin-1 and IL-12

**Figure 4:**
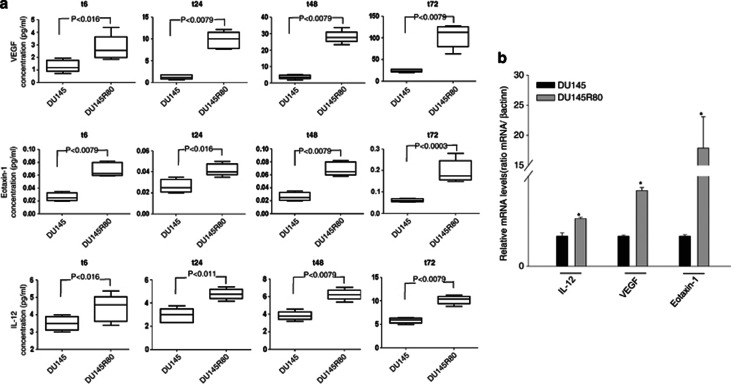
Increased expression of VEGF, Eotaxin-1 and IL-12 in DU145R80 compared with DU145 cells. (**a**) VEGF, Eotaxin-1 and IL-12 concentrations were evaluated in the culture media from DU145 and DU145R80 cells at the indicated time points after seeding by a multiplex ELISA-based immunoassay assay, and were plotted with box-and-whisker graphs. The boxes extend from the 25th to the 75th percentile, and the line in the middle is the median. The error bars extend down to the lowest value and up to the highest. (**b**) IL-12, VEGF and Eotaxin-1 mRNA expression was evaluated by RT-PCR after 6 h of cell culture. The data are representative of at least three independent experiments, include the means±S.D. of technical triplicates and reported statistical analysis of DU145R80 *versus* DU145 cells (*P<0.001, IL-12 and VEGF; *P*=0.014, Eotaxin-1)

An increased number of secreted factors produced by tumour cells or the tumour microenvironment may contribute to EMT and a more aggressive tumour phenotype. In this regard, we further characterised DU145R80 cells by studying the expression of about 30 cytokines, chemokines and angiogenic factors secreted in the culture medium using a multiplex ELISA assay. As shown in Figure 4a, a statistically significant higher secretion of VEGF, Eotaxin-1 and IL-12 was demonstrated in DU145R80 cells compared with parental DU145 cells, and this result was confirmed by a concomitant increase in gene expression (Figure 4b). These data suggest that the increased production of cytokines, such as Eotaxin-1 and IL-12 and proangiogenic factors, such as VEGF, could contribute at least in part, to the aggressive and invasive phenotype in our ZOL-resistant model.

### Activation of p38-MAPK is responsible for the acquisition of resistance to ZOL in DU145R80 cells

We and others have previously identified p38-MAPK as critically involved in the sensitivity of cancer cells to the antitumour effect of ZOL. ^12, 17, 18^ (Bruzzese *et al.*, paper submitted). In this regard, we next evaluated, the activation of p38-MAPK in both sensitive and resistant cells, to verify whether the p38-MAPK pathway can also be involved in the acquired ZOL resistance of DU145R80 cells.

**Figure 5:**
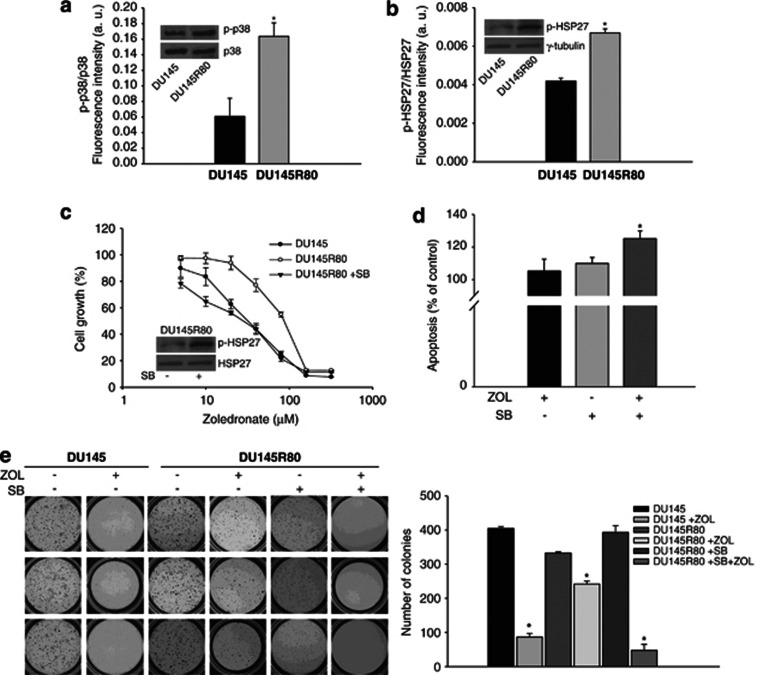
P38-MAPK activation was involved in the resistance to ZOL of DU145R80 cells. (**a**) Analysis of p38-MAPK and HSP27 activation evaluated by a phosphoprotein ELISA-based immunoassay (see Materials and Methods) (**a** and **b**) and by western blot (**a** and **b** inset panels). Values from phosphoprotein assay are means±S.D. of two independent experiments performed in triplicates and statistical analysis of DU145R80 *versus* DU145 cells is reported (*P=0.037, p38-MAPK; *P*=0.005, HSP27). (**c**) DU145R80 cells were treated for 96 h with increasing concentrations of ZOL alone or after 24 h of pretreatment with SB 30 *μ*ℳ and compared with DU145 treated with ZOL alone. Cell growth expressed as percentage of control was assessed by sulforhodamine B colorimetric assay (see Materials and Methods) and each point is the mean±S.D. of three independent experiments. Expression of p-HSP27 and HSP-27 in DU145R80 cells untreated or treated with SB 30 *μ*ℳ for 24 h were evaluated by western blot (inset panel). (**d**) Apoptosis evaluated by AnnexinV binding and cytofluorimetric analysis in DU145R80 cells untreated or treated with ZOL 20 *μ*ℳ alone or in combination with SB 30 *μ*ℳ for 48 h. Values of apoptotic cells are means±S.D. of three independent experiments and were expressed as per cent of untreated cells (100%). Statistical analysis demonstrated significant differences only in ZOL+SB combination *versus* untreated cells (*P=0.026). (**e**) Soft-agar clonogenic assay was performed on DU145 and DU145R80 cells untreated or treated with ZOL alone (20 *μ*ℳ) or in combination with SB (30 *μ*ℳ) for 21 days, in 24-well plates. Colonies >100 *μ*m were scored by a colony counter. Left: images from a representative experiment; right: values expressed as number of colonies are means±S.D. from at least two independent experiments performed in triplicates. Statistical analysis results are reported (*P<0.001, ZOL *versus* untreated cells in DU145; *P*=0.005, ZOL *versus* untreated cells in DU145R80; *P*=0.002, *P*=0.005, *P*=0.003, ZOL+SB combination *versus* untreated, *versus* ZOL or *versus* SB-treated cells, respectively, in DU145R80)

As shown in Figure 5a, DU145R80 showed a clear increase in p38-MAPK phosphorylation compared with DU145 cells, a finding that was confirmed by western blot analysis (Figure 5a inset), whereas no major differences in other MAP kinases, such as ERKs, were demonstrated (data not shown). We next assessed whether increased phosphorylation of p38-MAPK resulted in increased protein activity in DU145R80 cells. Both phosphorylation by ELISA assay and western blot analysis showed an increase in the phosphorylation status of heat shock protein-27 (HSP27), a confirmed p38 target (Figure 5b, inset). To further investigate the involvement of p38-MAPK in the ZOL resistance mechanism, we next adopted a pharmacological approach to inhibit p38-MAPK activity using the specific inhibitor SB203580 (SB). As shown in Figure 5c, by treating DU145R80 cells with SB 24 h beforehand and then concomitantly with ZOL, we demonstrated a complete reversion of the resistance with an IC_50_ value of ZOL (28 *μ*ℳ), similar to that of the parental DU145 cell line (IC_50_=32 *μ*ℳ).

**Figure 6:**
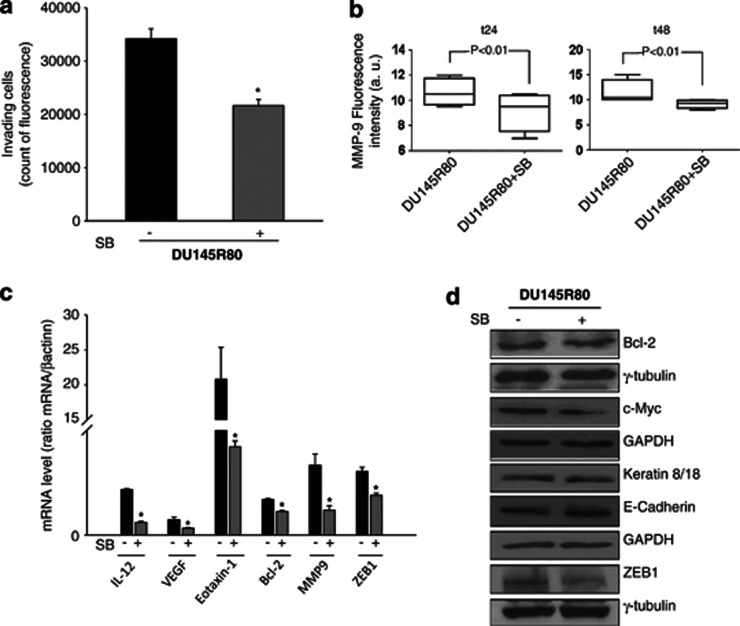
P38-MAPK activation regulated invasion, EMT and the expression of Bcl-2, c-Myc, MMP9 and cytokines in DU145R80. (**a**) Invasion capability of DU145R80 cells untreated or treated with SB (30 *μ*ℳ) for 24 h was evaluated as the ability to invade matrigel coated chambers (see Materials and Methods). Values are means±S.D. from three independent experiments performed in duplicates and statistical analysis is reported (*P<0.001). (**b**) MMP-9 concentration was evaluated in the culture media of DU145R80 cells untreated or treated with SB (30 *μ*ℳ) for the indicated time points after seeding, by a multiplex ELISA-based immunoassay assay, and were plotted with box-and-whisker graphs. The boxes extend from the 25th to the 75th percentile and the line in the middle is the median. The error bars extend down to the lowest value and up to the highest. (**c**) IL-12, VEGF, Eotaxin-1, Bcl-2, MMP9 and ZEB1 mRNA expression was evaluated by RT-PCR in DU145R80 cells untreated or treated with SB (30 *μ*ℳ) for 6 h. Values are means±S.D. from three independent experiments performed in duplicates and statistical analysis is reported (*P<0.01). (**d**) Western blot analysis of Bcl2, c-Myc, Keratin 8/18, E-cadherin and ZEB1 proteins expression evaluated in DU145R80 cells untreated or treated with SB (30 *μ*ℳ) for 72 h of cell culture. GAPDH and *γ*-tubulin were used as protein loading control

We confirmed the block of p38-MAPK activity in treated cells, showing that SB could inhibit activation of the downstream effector HSP27 (Figure 5c, inset). Moreover, we demonstrated that pharmacological blockade of p38-MAPK determined in DU145R80 cells resulted in a clear augmentation of apoptosis in combination with ZOL-treated compared with untreated cells, whereas treatment with ZOL or SB alone showed no statistically significant increase in the rate of apoptosis (Figure 5d). Interestingly, as shown in Figures 6b and c, the resistant cell line treated for 24 h with SB also showed a reduction in both the protein and gene expression levels of Bcl-2, suggesting that p38-MAPK activation could, at least in part, rescue DU145R80 cells from apoptosis by a mechanism involving Bcl-2.

Finally, as shown in Figures 5e and a marked reduction in the number of DU145R80 colonies was demonstrated in the ZOL-SB combination treatment compared with untreated cells under anchorage-independent conditions.

### Inhibition of p38-MAPK reduced the aggressive features of DU145R80 cells

We next investigated the role of p38-MAPK in the regulation of invasion and demonstrated that DU145R80 cells in the presence of the p38-MAPK inhibitor showed a marked reduction in the ability to invade a three-dimensional extracellular matrix compared with untreated cells (Figure 6a). Moreover, because we found that DU145R80 cells expressed a higher level of MMPs compared with the parental cell line, we investigated the effect of SB on MMP-9 expression. A clear reduction in both MMP-9 protein secreted in the culture medium and gene expression was observed in SB-treated cells (Figures 6b and c), suggesting that activation of p38-MAPK signalling in DU145R80 ZOL-resistant cells may regulate cell invasion by influencing metalloproteinase expression.

Furthermore, we observed that EMT, as well as cytokines and proangiogenic factors could also be regulated by p38-MAPK in DU145R80 cells. In fact, an increase in E-cadherin and keratin 8/18 proteins paralleled by a concomitant reduction in ZEB1 mRNA and protein, was detected in SB-treated compared with untreated cells (Figures 6c and d). Similarly, IL-12, Eotaxin-1 and VEGF mRNA expression was clearly downregulated by SB. A slight reduction in c-Myc protein expression was also observed in DU145R80 cells after treatment with SB (Figure 6d).

All together, these data suggest a critical role of the p38-MAPK pathway in both ZOL resistance and aggressive features of DU145R80 cells.

## Discussion

In the present study, we selected and characterised the resistant cell line DU145R80 established from DU145 PCa cells after 6 months of treatment with increasing concentrations of ZOL. Thus far, only ZOL-resistant breast cancer^11^ and osteosarcoma^12, 13^ cell lines have been established, and to our knowledge, the DU145R80 cell line is the first established ZOL-resistant PCa model. The mechanism of resistance appeared to be specific for N-BPs because a clear cross-resistance was observed against pamidronate but not *versus* clodronate, as also demonstrated by Ory *et al.*^13^ in osteosarcoma ZOL-resistant cells.

A strong resistance compared with parental DU145 cells was confirmed by the capability of DU145R80 cells to grow in soft agar under ZOL-treated conditions. Notably, DU145R80 cells, compared with DU145 cells, were resistant to spontaneous apoptosis under both adherent and anoikis conditions and acquired an increased invasive capability, suggesting that an extensive ZOL treatment could contribute to more aggressive features in PCa cells. The increased invasive capability of DU145R80 cells was paralleled by the increase in MMP-2/9 gene expression and secretion in the culture media, a critical event that has been demonstrated to support invasion and metastasis in PCa.^19^ Interestingly, we have previously shown in a phase I trial that a decreased serum level of MMP-2/9 correlated with disease control in hormone-refractory PCa patients undergoing docetaxel and ZOL combination treatment, whereas increased serum levels of both MMPs were observed in non-responding patients.^20^

Furthermore, we demonstrated that DU145R80, compared with parental DU145 cells, undergo EMT, a key process required during embryonic development that is associated with a tumour initiating/stem-like phenotype and that occurs in, and may contribute to, PCa progression and metastasis.^21^ In particular, we found that DU145R80 reduced the expression of the homotypic cellular adhesion molecule E-cadherin, the main molecular alteration involved in EMT^21^ by upregulating the expression of its suppressor ZEB1. In addition, DU145R80 cells showed downregulation of other epithelial markers, such as keratin 8/18, and upregulation of the mesenchymal marker vimentin. Moreover, the EMT feature of DU145R80 cells is also supported by the reduction of the growth rate of these cells compared with parental DU145 cells because EMT-inducing factors have been shown to reduce cell proliferation.^22^ Recent studies have confirmed that transient EMT is critical for circulating tumour cell survival and dissemination.^16^ In agreement with these observations, the marked reduction in E-cadherin expression observed in DU145R80 cells and the acquisition of EMT features are probably also responsible for the ability of DU145R80 cells to survive under conditions that reproduce the bloodstream, such as anoikis conditions.

Among the factors suspected to control PCa, incidence and progression, cytokines, chemokines and their receptors, produced by tumour cells and by the stromal microenvironment, exert pleiotropic actions, including the modulation of growth, angiogenesis, invasion, metastasis and hormone escape.^3, 23^ We demonstrated that DU145R80 cells, compared with DU145 cells, showed increased expression and secretion of VEGF, a proangiogenic factor that may contribute to the induction of EMT in tumour cells and that is described as overexpressed during the transition from prostate intraepithelial neoplasia (PIN) to invasive carcinoma.^24^ In addition to VEGF, among several cytokines and chemokines evaluated in the conditioned media of DU145R80 cells compared with DU145 cells, we observed a significant increased expression of only IL-12 and Eotaxin-1. IL-12, a well-known mediator of the immune response produced mainly by dendritic cells, has been described to exert an antitumour effect;^25^ however, its role in tumour cells is less clear, and additional studies are ongoing to further investigate this finding. Conversely, Eotaxin-1/CCL11, a CC-type chemokine and a potent chemotactic factor for eosinophils, is associated with disease progression in cancer patients and has been recently found at significantly higher levels in the serum of men with PCa than in that of men with benign prostate hypertrophy or no evidence of prostatic disease.^26^ At the molecular level, we showed that DU145R80 cells, relative to parental DU145 cells, were characterised by overexpression of the anti-apoptotic protein Bcl-2 and the oncoprotein c-Myc, both of which can be implicated, at least in part, in the mechanism of ZOL resistance. In agreement with this finding, Kars *et al.*^11^ demonstrated that an increased Bcl-2/Bax expression ratio correlated with ZOL resistance developed in MCF-7 breast cancer cells. Conversely, our findings further support the view of an aggressive phenotype in DU145R80 cells because Bcl-2 expression was significantly upregulated in PCa with an aggressive phenotype, as indicated by the correlation with a high Gleason score, advanced stage and high proliferation index.^27^ However, c-Myc appears to be activated at the earliest phases in prostatic intraepithelial neoplasia, contributing to disease initiation and progression, and c-Myc is a key precursor lesion for invasive prostatic adenocarcinoma.^28, 29^

Ory *et al.*,^13^ dissecting the mechanism of ZOL resistance in osteosarcoma cells, indicated that prolonged treatment with ZOL increased the expression of FPPS, a critical enzyme in the mevalonate pathway that is inhibited by N-BPs, leading to a resistant phenotype. However, in DU145R80 cells, we did not find a clear increase of FPPS compared with DU145 parental cells (data not shown).

Rather, the key finding of our study is the observation that both the acquired resistance to ZOL and the acquisition of the more aggressive phenotype are mediated by p38-MAPK activation. In detail, we showed by ELISA assay and western blotting strong activation of both p38-MAPK and its downstream effector HSP27 in DU145R80 cells compared with DU145 cells.

The p38-MAPK pathway is implicated in the regulation of many aspects of cell physiology and has a role in several human diseases, including cancer.^30^ In particular, many studies have demonstrated that the p38-MAPK pathway antagonises cell proliferation and regulates proapoptotic signalling; accordingly, several negative regulators of p38-MAPK have been found to be overexpressed in human tumours and cancer cell lines.^31^ Conversely, increased activation of p38-MAPK has been correlated with malignancy in several types of cancers, including PCa, and several lines of evidence have suggested that, in cancer cells, p38-MAPK activation may facilitate proliferation, survival, invasion and drug resistance.^31^ Interestingly, Dunford *et al.*^32^ demonstrated in macrophages and osteoclasts that potent N-BPs, including ZOL, as well as other inhibitors of the mevalonate pathway, by inhibiting protein prenylation, cause inappropriate and sustained activation, rather than inhibition, of some small GTPases, such as RAC, and in turn increase p38-MAPK activity. Interestingly, the increased p38-MAPK activation was not involved in N-BP-induced apoptosis but rather partially suppressed the proapoptotic effect of N-BPs in these cells. We have recently reported that Cyr61, a membrane-bound integrin interactor protein, is a resistance factor to the proapoptotic effect of ZOL in androgen-independent PCa cells.^33^ Interestingly, cross-talk between Cyr61 activation and p38-MAPK has been reported elsewhere.^34, 35^ A potential role of p38-MAPK activation and/or of its downstream target HSP27 in the resistance to N-BP treatment has also been previously proposed in breast cancer^17^ and osteosarcoma^12, 18^ preclinical models. Moreover, we have recently demonstrated that treatment with ZOL increased p38 phosphorylation in several PCa cells (Bruzzese *et al.*, paper submitted).

In the present study, coadministration of ZOL with the SB specific inhibitor of p38-MAPK reverted ZOL resistance in DU145R80 cells in both anchorage-dependent and anchorage-independent proliferation assays, as well as apoptosis assays. Moreover, we also observed a partial decrease in both Bcl-2 and c-Myc protein levels after SB administration, and thus, we conclude that the p38-MAPK pathway confers resistance to apoptosis, at least in part, through the regulation of both Bcl-2 and c-Myc expression. Similarly, Phong *et al.*^36^ demonstrated a prosurvival role of p38-MAPK during the G(2) DNA damage checkpoint response through upregulation of the Bcl-2 family proteins and c-Myc.

Interestingly, migration and invasion have been previously linked in PCa cells to p38-MAPK-dependent modulation of MMP-2 and MMP-9.^37^ In this regard, by treating DU145R80 cells with the SB inhibitor, we also reduced the invasion capability, expression and secretion of MMP-9. In addition, SB reactivated the expression of E-cadherin and, in part, keratin 8/18 and downregulated ZEB1 gene and protein expression, suggesting a regulatory function of the p38-MAPK pathway regarding EMT status in DU145R80 cells. Finally, we demonstrated in SB-treated DU145R80 cells significantly reduced expression of VEGF, IL-12 and Eotaxin-1, which was overexpressed in ZOL-resistant compared with parental DU145 cells. The expression of all these factors has been previously found to be regulated by p38-MAPK activation and has been correlated with angiogenesis, inflammation and the immune response, frequently in non-cancer cells.^30, 38, 39^

On the other hand, VEGF as well as both IL-12 and Eotaxin-1 has been described to regulate p38-MAPK activity after binding with their specific receptors, and p38-MAPK pathway engagement was demonstrated to be critical for their effect on target cells.^30^ Thus, we could not exclude the possibility that an autocrine pathway is activated by the increased secretion of these cytokines in DU145R80 cells, which, in turn, stimulates p38MAPK and regulates all or some of the features that we have described above. Some experiments are currently ongoing to verify this hypothesis.

**Figure 7:**
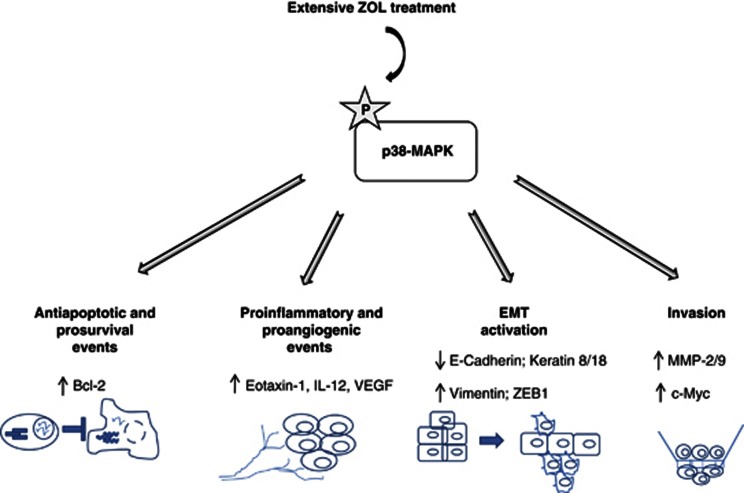
Proposed model for the mechanisms underlying the acquired resistance to ZOL and the parallel acquisition of an aggressive phenotype mediated by p38-MAP Kinase activation

In conclusion, this study demonstrated, for the first time, that prolonged treatment of PCa cells with ZOL was responsible for the activation of the p38-MAPK, which has a central role in the regulation of several biological process, such as antiapoptotic, prosurvival, proinflammatory and proangiogenic events, as well as EMT activation and invasion (Figure 7). The direct correlation between all the proteins regulating the above events and p38-MAPK was also highlighted by a protein interatomic analysis that was performed using Ingenuity software (Supplementary Figure 2). This protein network could have a critical role to confer ZOL resistance and a more aggressive phenotype to PCa cells. Moreover, this analysis may allow more detailed examination of the molecular mechanisms and could help to plan combination drug treatments aiming to overcome or prevent these occurrences.

## Materials and Methods

### Materials

Clinical grade ZOL; Zometa was provided by Novartis Pharma (Basel, Switzerland). The stock was prepared in distilled water and diluted to appropriate concentrations in culture medium before addition to the cells. Pamidronate, clodronate and the p38-MAPK inhibitor pyridinyl imidazole SB203580 (SB) were obtained from Calbiochem (San Diego, CA, USA).

The primary antibodies c-Myc, Bcl-2, phospho-pP38-MAPK and total p38-MAPK, phospho-HSP27 and total HSP27, keratin 8/18 and TCF8/ZEB1, were purchased from Cell Signaling Technology (Boston, MA, USA); *γ*-tubulin and GAPDH, from Santa Cruz Biotechnology Inc. (San Jose, CA, USA); N-cadherin, from Abcam (Cambridge, England); vimentin, from Dako-Citomation (Glostrup, Denmark). Secondary antibodies anti-rabbit, anti-goat and anti-mouse were purchased from Abcam. Sulphorhodamine B (SRB) was purchased from ICN Biomedicals (Irvine, CA, USA); all media, serum, antibiotics and glutamine were purchased from Cambrex Bio Science (Verviers, Belgium). Foetal bovine serum was purchased from Gibco (GIBCO Life Technologies Italia Fil, Life Technologies Europe BV, Monza, Italy). AnnexinV-FITC was purchased from Becton Dickinson (San Jose, CA, USA).

### Cell culture and ZOL-resistant cell selection

The PCa cell line DU145 was purchased from American Type Culture Collection (Rockville, MD, USA). ZOL-resistant DU145R80 cells were obtained by treating DU145 with increasing concentrations of ZOL, starting from 17 *μ*ℳ up to 80 *μ*ℳ. After 6 months, the selected cells were ‘pooled', in order to avoid clonality, and were tested to evaluate the rate of drug resistance, calculated as the resistance index, RI=IC_50_DU145R80/IC_50_DU145. DU145 and DU145R80 cells were grown in RPMI 1640 medium containing 10% heat inactivated FBS, 50 units/ml penicillin, 500 *μ*g/ml streptomycin, 20 mℳ Hepes (pH 7.4) and 4 mℳ glutamine. The cells were grown in a humidified atmosphere composed of 95% air and 5% CO_2_ at 37 °C. Suspension culture was performed in Ultra-low attachment flasks (Corning Incorporated Life Sciences, Tewksbury, MA, USA).

### Cell proliferation assay

Cell viability was measured using SRB after 96 h of treatment in 96-well plates, as described previously.^40^

### Clonogenic agar assay

Cells were plated in 24-well flat-bottomed plates using a two-layer soft agar system, as previously described.^40^ After 3 h, the cells were treated with ZOL and/or SB at the indicated concentrations. Medium (with or without drugs) was replaced every 3 days.

Colonies grew for 14 or 21 days and then were stained overnight with NBT (nitroblue-tetrazolium), photographed, analysed and counted using Image-Pro-Plus (Immagini and Computer snc Bareggio, Milano, Italy). Colonies >100 *μ*m were scored as positive. All the experiments were performed in triplicate.

### Apoptosis assays under adherent and non-adherent (anoikis) cell culture conditions

DU145 and DU145R80 cells were cultured in adhesion in 60-mm cell culture dishes or under non-adherent conditions using specific ultralow-attachment flasks T25 (Corning Life Sciences). After 24–48–72 h for adherent conditions and 48–72–96 h for suspension culture, apoptosis was tested using AnnexinV staining. AnnexinV binding was identified by flow cytometry using an AnnexinV-FITC staining procedure following the manufacturer's instructions (Becton Dickinson).

### Invasion assay

Cell invasion was measured using a Boyden modified assay using transwell chambers (Costar, Cambridge, MA, USA) with 8-mm pore polycarbonate filters that were coated with 50 mg/ml of Matrigel (BD Biosciences, Franklin Lakes, NJ, USA) and diluted in serum-free medium, as previously described.^41^ The experiments were performed in triplicate.

### Protein extraction and western blotting

Cells grown and treated as indicated were collected, lysed and separated on SDS polyacrylamide gel electrophoresis gels, and then proteins were transferred to nitrocellulose membranes, immunoblotted with specific antibodies and probed with the appropriate horseradish peroxidase-linked IgG, as described elsewhere.^40^ Immunoreactive bands were detected by enhanced chemiluminescence (GE Healthcare, Milan, Italy).

### RNA isolation and quantitative RT-PCR assay

Total RNA was isolated using the RNeasy plus mini kit (Qiagen, Hilden, Germany), as indicated by the manufacturer's instructions. The NanoVue Plus Spectrophotometer (GE Healthcare) was used to quantify RNA. The quality and integrity of the RNA were confirmed by agarose gel electrophoresis and ethidium bromide staining, followed by visual examination under UV light. Reverse Transcription was performed using the QuantiTect Reverse Transcription Kit (Qiagen). QuantiTect Primer Assays (Qiagen) were used to quantify RNA levels of bcl-2 (Hs_BCL2_1_SG), ZEB1 (Hs_ZEB1_2_SG), MMP-2 (Hs_MMP2_1_S4), MMP-9 (Hs_MMP9_1_S4), vimentin (Hs_VIM_1_SG), E-cadherin (Hs_CDH1_1_SG) and *β*-actin (Hs_ACTB_2_SG). Each sample was assayed in quadruplicate with 20 ng of input RNA per well in a 25-*μ*l reaction volume containing 1 × QuantiTect SYBR Green PCR Master Mix and 1 × QuantiTect gene expression assay (Qiagen). The specificity of the produced amplification product was confirmed by examination of dissociation reaction plots, and the resulting PCR products were run on 2% agarose gels to confirm that the correct molecular sizes were present. Cycle threshold values (*C*_t_) generated using Sequence Detection System 2.2.2 (Applied Biosystems, Foster City, CA, USA) default parameters were exported to determine relative mRNA abundances among genes in the classifier. Gene expression was normalised to *β*-actin expression. Taqman probes were used to quantify RNA levels specific for VEGF A (VEGF_Hs00900055_m1), IL-12 (Hs00168405_m1), Eotaxin-1 (Hs00237013_m1) HU ACTB (Hs01060665_g1) (Applied Biosystems). Each sample was tested in triplicate using RT-PCR and the ABI Prism 7900 HT Sequence Detection System (Applied Biosystems) and three independent experiments were used to quantify relative gene expression.

### Bio-Plex multiplex assay for phosphoproteins, cytokines and MMPs

A multiplex biometric ELISA-based immunoassay, the Luminex platform (Bio-plex, Bio-Rad Lab Inc., Hercules, CA, USA), containing dyed microspheres conjugated with a monoclonal antibody specific for a target protein was used, according to the manufacturer's instructions, to evaluate the trends and concentrations of different soluble molecules in cellular lysates or culture supernatants. The levels of protein phosphorylation were determined on protein lysates from cells cultured and treated as indicated. The targeted phosphorylated proteins that were assayed included p38-MAPK (Thr180/Tyr182), HSP27 (Ser78), Erk1/2 (Thr202/Tyr204) and JNK (Thr183/Tyr185). The phosphoprotein fluorescence intensities were normalised to the total protein expression intensities. Data acquisition and analysis were performed using Bio-Plex Manager software 3.0 (Bio-Rad).

The levels of cytokines, chemokines and growth factors were measured using a Human Cytokine 30-Plex Panel (Invitrogen, Corporation, Camarillo, CA, USA). In particular, the following cytokines were evaluated: EGF, Eotaxin-1, FGF basic, G-CSF, GM-CSF, HGF, IFN-*α*, IFN-*γ*, IL-1ra, IL-1*β*, IL-2, IL-2r, IL-4, IL-5, IL-6, IL-7, IL-8, IL-10, IL-12 (p40/p70), IL-13, IL-15, IL-17, IP-10, MCP-1, MIG, MIP-1*α*, MIP-1 *β*, RANTES, TNF-*α* and VEGF. Basal expression of MMP-2/9, or MMP-2/9 expression after treatment with SB, was evaluated using the Milliplex MAP system (Merck-Millipore, Darmstadt, Germany). A quintuplicate of each sample was analysed. Levels of all cytokines or MMP-2/9 were determined using a Bio-Plex array reader (Luminex, Austin, TX, USA). Data from experiments on cytokines were analysed using the Bio-Plex Manager software, version 3.0 (Bio-Rad). Sample concentrations were immediately interpolated from the standard curves. The single values were considered elevated when results were higher than the mean +2 S.D. of the controls. Regression analysis was performed to derive an equation that was then used to predict the concentration of these proteins in the cell supernatant samples. The nonparametric Mann–Whitney *U*-test was used to evaluate differences between cytokine and MMP factor ratios in DU145 and DU145R80 cells.

### Statistical analysis

The results of *in vitro* cell proliferation and clonogenic agar assays are expressed as the means for at least three independent experiments performed in triplicate or quadruplicate (bar, S.D.). Similarly, the results of apoptosis by flow cytometry analysis or invasion assays are expressed as the means for at least three independent experiments (±S.D.). The RT-PCR data for mRNA expression are representative of at least three independent experiments and include the means±S.D. of technical triplicates. The Bio-Plex data are representative of at least two independent experiments and include the means±S.D. of technical quadruplicates or quintuplicates. Statistical significance was proved by two-sided Student's *t*-tests (normal distribution), and all statistically significant *P*-values (<0.05) are reported in the manuscript or in figure legends. Representative results from western blotting from a single experiment are presented; additional experiments yielded similar results. All statistical evaluations were performed using Sigma Stat software (Systat Software Inc., San Jose, CA, USA).

Interactome pathway analysis was performed using Ingenuity Pathway Analysis 7.1 (Ingenuity Systems Inc., Redwood City, CA, USA).

## Abbreviations

CRPC: androgen refractory metastatic prostate cancer
EDTA: Ethylenediaminetetraacetic acid
*ELISA*: Enzyme-linked immunosorbent assay
EMT: epithelial to mesenchymal transition
*ERKs*: extracellular-signal-regulated kinases
FPP: farnesylpyrophosphate
FPPS: farnesyl diphosphate synthase
GGPP: geranylgeranyl pyrophosphate
HSP27: heat shock protein-27
IL-12: interleukin 12
MAPK: mitogen-activated protein kinase
MMP-2: metalloproteinase-2
MMP-9: metalloproteinase-9
N-BPs: Nitrogen-containing bisphosphonates
NBT: nitroblue-tetrazolium
PCa: Prostate cancer
PIN: prostate intraepithelial neoplasia
SDS-PAGE: SDS–polyacrylamide gel electrophoresis
VEGF: Vascular endothelial growth factor
ZOL: Zoledronic Acid
