# Supplementary figures and images for: Acquired resistance to zoledronic acid and the parallel acquisition of an aggressive phenotype are mediated by p38-MAP kinase activation in prostate cancer cells
Source: Cell Death Dis. 2013 May 23;4(5):e641–. doi: 10.1038/cddis.2013.165 (PMC3674372; doi:10.1038/cddis.2013.165)

## Slide 1
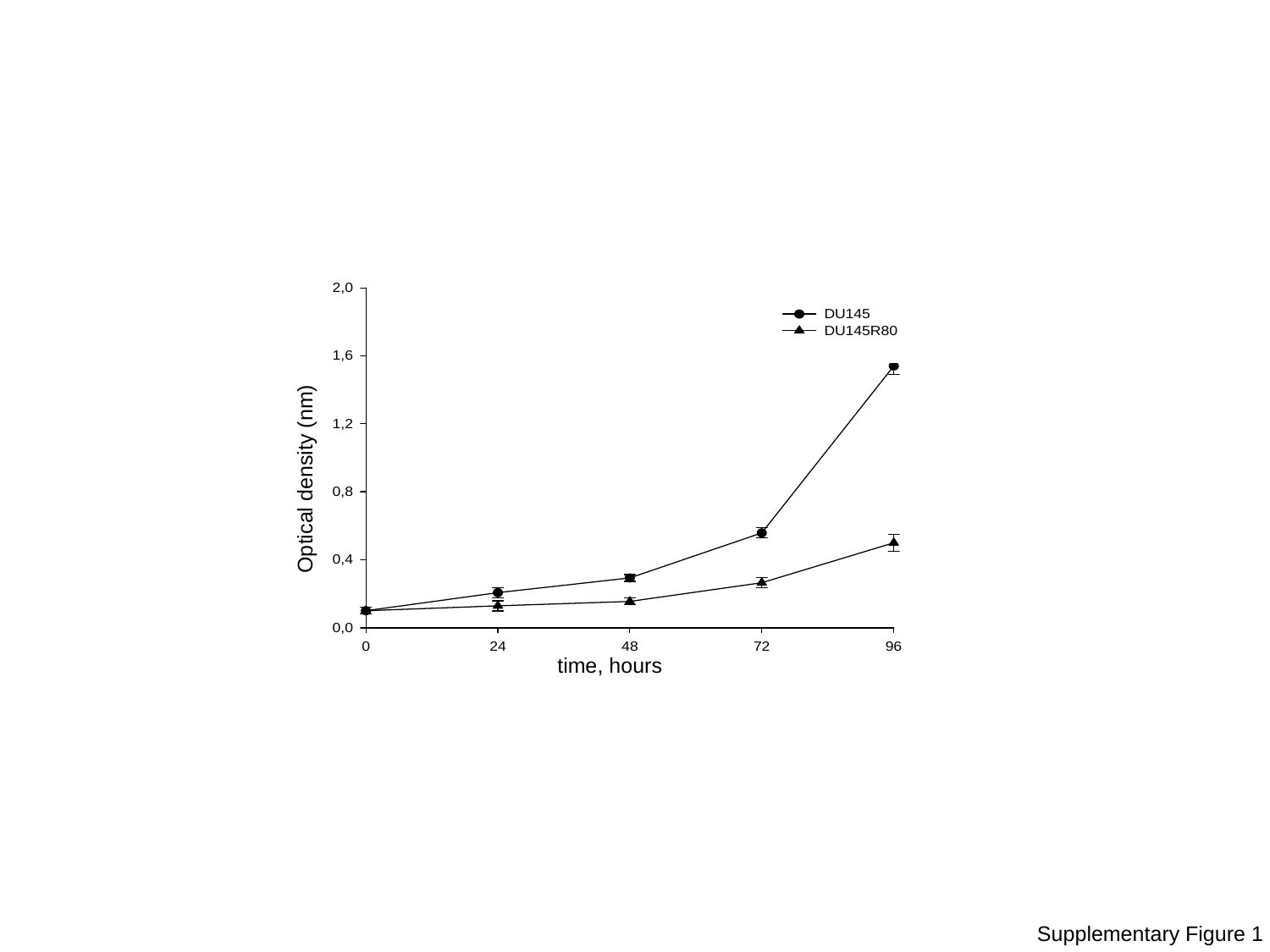

Optical density (nm)
time, hours
Supplementary Figure 1

Supplement: Supplementary Figure 1 [file cddis2013165x1.ppt]

## Slide 1
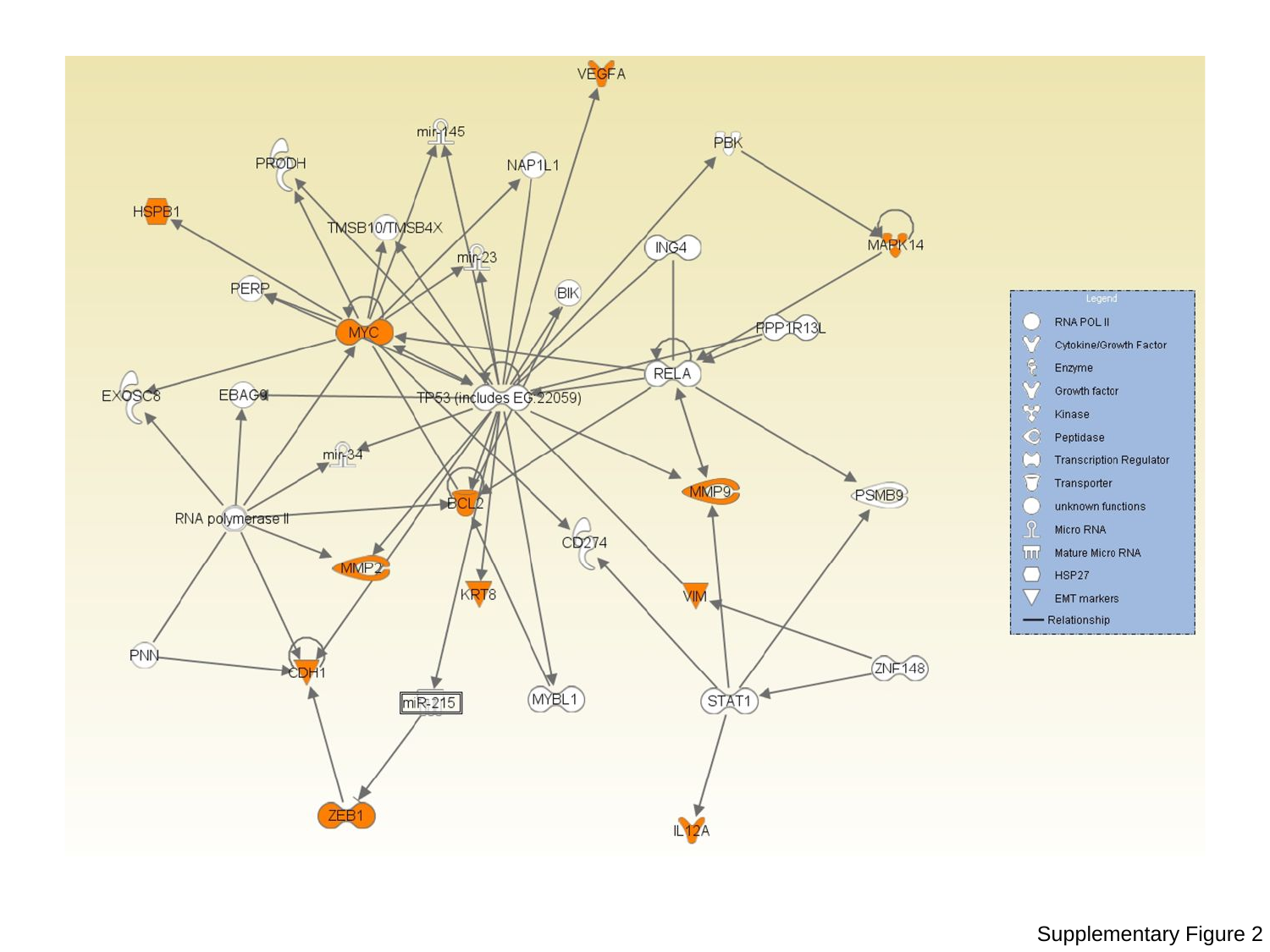

Supplementary Figure 2

Supplement: Supplementary Figure 2 [file cddis2013165x2.ppt]
