# Supplementary material for: Acquired resistance to zoledronic acid and the parallel acquisition of an aggressive phenotype are mediated by p38-MAP kinase activation in prostate cancer cells
Source: Cell Death Dis. 2013 May 23;4(5):e641–. doi: 10.1038/cddis.2013.165 (PMC3674372; doi:10.1038/cddis.2013.165)
Supplement: Supplementary Information [file cddis2013165x3.doc]

**LEGENDS TO SUPPLEMENTARY FIGURES**

**Supplementary Figure 1.** Cell growth of DU145 and DU145R80 cells was assessed at the indicated time points by sulforhodamine B colorimetric assay. Values are the mean ± SD from at least three independent experiments performed in quadruplicates.

**Supplementary Figure2.** Interactomic pathway analysis of experimental data was performed using Ingenuity Pathway Analysis (IPA) of significant molecules. The graph shows the closely associated network for analyzed proteins (evidenced with orange symbols) and other molecules (evidenced with white symbols).
